# Loss of HIV-infected patients on potent antiretroviral therapy programs in Togo: risk factors and the fate of these patients

**DOI:** 10.11604/pamj.2013.15.35.2198

**Published:** 2013-05-26

**Authors:** Bayaki Saka, Dadja Essoya Landoh, Akouda Patassi, Stephane d'Almeida, Assetina Singo, Bradford D Gessner, Palokinam Vincent Pitché

**Affiliations:** 1 Service de dermatologie et infections sexuellement transmissibles, Centre Hospitalier Universitaire Sylvanus Olympio, Lomé, Togo; 2 Division de l’épidémiologie, Ministère de la Santé, Lomé, Togo; 3 Service des maladies infectieuses, Centre Hospitalier Universitaire Sylvanus Olympio, Lomé, Togo; 4 Programme national de lutte contre le SIDA et les IST (PNLS/IST), ministère de la Santé Togo; 5 Agence de Médecine Préventive, Paris, France

**Keywords:** Africa, HIV, human immunodeficiency virus, lost to follow-up, people living with HIV, Togo

## Abstract

**Introduction:**

National programs are facing challenges of loss to follow-up of people living with HIV/AIDS (PLWHA) on antiretroviral therapy (ART). We sought to identify risk factors associated with early loss to follow-up among HIV-infected patients on ART in Togo and the outcome of such patients.

**Methods:**

This was a retrospective cross-sectional study using medical records of all patients older than age 15 years enrolled at 28 treatment centers who were on ART programs and who were lost to follow-up from 2008 to 2011.

**Results:**

Of the 16,617 patients on ART, 1,216 (7.3%) were lost to follow-up. Most (94.1%) were infected with HIV-1 and 32.6% were in WHO stage III or IV. The median CD4 count was 118/mm3 (IQR: 58-178 cells/mm3). No telephone number was mentioned in the medical records of 212 patients. Of the 1004 patients whose phone number was listed, 802 patients (79.9%) were not reachable on the recorded number, 114 patients (11.4%) were alive and 88 patients (8.8%) had died. In multivariate analysis, factors associated with loss to follow-up during the first 6 months of ART were: age below 35 years (OR = 1.6; 95%CI: 1.2-2.2), female sex (OR = 1.8; 95%CI: 1.3-2.5), WHO stage III or IV (OR = 1.7; 95%CI: 1.3-2.2), existence of an opportunistic infection (OR = 2.3; 95%CI: 1.5-3.1), and follow-up in a public centre (OR = 1.9; 95%CI: 1.2-3.3).

**Conclusion:**

This study identified several factors associated with lost to follow-up during the first 6 months of ART, and confirmed high mortality among these patients. The National AIDS Program should strengthen medical support of PLWHA in Togo including active case follow-up.

## Introduction

Human immunodeficiency virus (HIV) infection and acquired immunodeficiency syndrome (AIDS) are major public health problems in sub-Saharan Africa. Since the introduction of antiretroviral access programs in Togo, significant progress has been noted in the care of people living with HIV (PLWHA). From November 2008, antiretroviral therapy (ART) was provided free of charge to PLWHA while biological monitoring became free in 2010. Since then, the number of PLWHA support centers has increased from 70 in 2008 to 115 in late 2010, scaling-up treatment coverage of antiretroviral therapy from 2% in 2004 to 62.8% in late 2010 [1].

In the framework of scaling up the treatment at the sub-national levels, national programs are facing several challenges including the problem of loss to follow-up of PLWHA following the start of ART in treatment centers [2–6]. The current study aimed to highlight factors associated with early loss to follow-up of HIV-infected patients in treatment centers in Togo and the outcome of such patients.

## Methods

This was a retrospective cross-sectional study carried out in 28 centers in Togo prescribing ART (two teaching hospitals, five regional hospitals, seven district hospitals/other public organizations, and 14 associations, NGOs, or accredited confessional structures). The choice of 28 treatment centers was done by convenience to cover the whole country, and targeted structures with large active patient populations. Indeed, over 80% of PLWHA in Togo are supported in these 28 centers. The study focused on medical records of PLWHA age older than 15 years on ART, who were lost to follow-up between January 2008 and October 2011. This period was selected because ART and biological screening became available for free during 2008 and 2011 was the last year of data available. According to the National AIDS control guidelines, a patient on ART was qualified as lost to follow-up when review of the medical records for the patient did not find evidence that he had died or moved four months after the last visit.

Once enrolled in a specific clinic, HIV-infected patients are requested to get all future treatment — provided at no charge — at that same clinic each month. Their contact numbers (if available) and information related to their clinic visits are recorded in their clinic record. To identify the outcome of patients lost to follow-up, study investigators called the patient's telephone number listed in the clinic record or his attendant's telephone number if this existed in the record. Patients were classified into two groups with respect to the time between the beginning of ART and loss of follow-up: early (less than or equal to 6 months) and late (more than 6 months).

Data were collected using a form validated by the Togolese National AIDS Program that had been tested in five clinics in Lomé. Six teams were deployed for data collection. Each team included two medical students who received two days of training on how to review patient's records in the HIV clinics; for each team, the two medical students reviewed the same patients’ records and then the study team reconciled inconsistencies in the information collected by individual abstractors

Data were recorded using the software Epi Info version 3.5.1. For continuous variables, medians and interquartile ranges were calculated while for categorical variables we calculated proportions and respective 95% confidence intervals. Our main outcome variable was loss to follow-up at less than or equal to 6 months compared to loss to follow-up at greater than 6 months. The chi-square test or Fisher′s exact test were used when appropriate in bivariate analysis. Multivariate backwards stepwise logistic regression analysis was performed to identify independent risk factors for the dichotomous outcome lost to follow up or not lost to follow up. All variables significant during bivariate analysis at a p-value less than 0.05 and variables previously associated with lost to follow-up were included in the multivariate analysis. However, we excluded CD4 count as this is a component of the World Health Organization stage, and we used the latter as a measure of severity. We then removed variables in a stepwise fashion that were not significant at a p value > 0.1. We also assess for the level of significance of the likelihood ratio of the test. No interaction was identified for variables included in the model. Multivariate logistic regression analyses were performed using the method of MANOVA in Stata 11.

This study was approved by the Ministry of Health of Togo's board committee. Reference No 01/2011/MS/DGS/DSSP/PNLS-IST. We obtained verbal consent from study subjects that participated in the phone call.

## Results

From January 2008 to October 2011, 16,617 patients were placed on ART in the 28 included medical care structures. Among the total of 16,617 PLWHA, 1216 (7.3%) were lost to follow after the initiation of ART. Of these 1216, 290 had early loss to follow-up while 926 had late loss to follow-up. Baseline characteristics of the 1216 patients are summarized in Table 1.

**Table 1:** Base line characteristics of 1216 patients with human immunodeficiency virus infection on anti-retroviral therapy who were lost to follow-up; Togo, 2008-2011

Profile of patients	Total of patients (n= 1216)
Age in year: median (IQR)	32 (29 - 43)
Age between 25 and 49 years, n (%)	966 (79.4)
Females, n (%)	755 (62.1)
**Type of HIV, n (%)**	
HIV1	1144 (94.1)
HIV2	52 (4.3)
HIV1 + 2	20 (1.6)
**WHO clinical stage, n** (%)	
Stage I	435 (35.8)
Stage II	385 (31.6)
Stage III	261 (21.5)
Stage IV	135 (11.1)
**CD4 count (cells/mm3), median (IQR)**	119 (55 - 188)
< 50 cells/mm3, n (%)	211 (17.4)
50-200 cells/mm3, n (%)	708 (58.2)
200-350 cells/mm3, n (%)	228 (18.7)
> 350 cells/mm3, n (%)	69 (5.7)
**Blood hemoglobin level**	
≤ 8g/dl, n (%)	946 (77.8)
> 8g/dl, n (%)	270 (22.2)
**Main therapeutic regimens, n (%)**	
2NRTIs* + 1NNRTI**	1030 (84.7)
2NRTIs* + 1PI***	127 (10.4)
3NRTIs*	59 (4.9)
**Opportunistic infection, CDC classification, n (%)**	**173 (14.2)**
Oropharyngeal candidiasis	42 (24.3)
Persistent vaginal candidiasis	30 (17.3)
Recurring zoster or involvement of an entire dermatome	9 (5.2)
Herpetic infection, chronic ulcer> 1 month	2 (1.2)
Pulmonary tuberculosis	23 (13.3)
Cerebral toxoplasmosis	10 (5.8)
Kaposi's sarcoma	10 (5.8)
Bacterial pneumonia/pneumocystosis (*Pneumocystis carinii*)	22 (12.7)
Infectious diarrhea	16 (9.2)
Cryptococcal meningitis	8 (4.6)
Molluscum contagiosum	1 (0.6)

* NRTI: Nucleoside Reverse Transcriptase Inhibitors

** NNRTI: Non-Nucleoside Reverse Transcriptase Inhibitors

*** PI: Protease Inhibitors

During bivariate analysis, of the 1216 people lost to follow-up, those with early compared to late loss to follow-up were more likely to be under age 35 years (RR 1.6; 95% CI: 1.3 - 2.0; p< 0.001), to have had an opportunistic infection at the beginning of ART (RR = 1.7; 95% CI: 1.4 - 2.1), to live in a rural area (RR = 1.6; 95%CI: 1.2 - 2.1; p < 0.001), and to have received treatment in a public structure (RR = 1.3; 95%CI: 1.1 - 1.9; p = 0.05) (Table 2).

**Table 2:** Factors associated with lost to follow-up (LFU) at ≤6 months compared to greater than 6 months among patients with human immunodeficiency virus (HIV) infection taking antiretroviral therapy (ART); Togo, 2008- 2011. (n = 1216)

Variables from medical records	LFU ≤ 6 months (N = 290)	LFU > 6 months (N = 926)	Chi-square	RR	95% CI	p-value
**Age**						
≤ 35 years	180 (62.1)	429 (46.3)	Ref. 22.3	Ref.1.6	Ref.1.3 - 2.0	< 0.001
> 35 years	110 (37.9)	497 (53.7)				
**Sex**						
Female	215 (74.1)	540 (58.3)	Ref.22.8	Ref.1.8	Ref. 1.4 - 2.2	< 0.001
Male	75 (25.9)	386 (41.7)				
**Type of HIV**						
HIV 1	278 (95.8)	866 (93.5)	Ref. 2.2	Ref. 1.4	Ref.0.9 – 2.8	0.14
HIV 1 + 2 / HIV 2	12 (4.2)	60 (6.5)				
**CD4 Count**						
≤ 50	76 (26.2)	135 (14.6)	Ref. 20	Ref. 1.6	Ref. 1.4 – 2.1	0.001
> 50	214 (73.2)	791 (85.5)				
**Blood hemoglobin level**						
> 8g/dl	70 (24.1)	200(21.6)	Ref.0.8	Ref.1.1	Ref. 0.8 - 1.4	0.18
≤ 8 g/dl	220 (75.9)	726 (78.4)				
**WHO clinical stage**						
Stage III or IV	120 (41.4)	276 (29.8)	Ref. 12.9	Ref. 1.5	Ref. 1.2 - 1.8	0.0003
Stage I or II	170 (58.6)	650 (70.2)				
**Existence of opportunistic infections**						
Yes	65 (22.4)	108 (11.7)	Ref. 20.1	Ref. 1.7	Ref. 1.4 - 2.2	< 0.001
No	225 (77.6)	818 (88.3)				
**Existence of side effects**						
Yes	10 (3.4)	44 (4.7)	Ref. 1.1	Ref. 0.8	Ref. 0.4 - 1.3	0.29
No	280 (96.6)	882 (95.3)				
**Residence**						
Rural	67 (30.2)	134 (18.7)	Ref. 13.2	Ref. **1.6**	Ref. 1.3-2.0	0.0002
Urban	155 (69.8	582 (81.3)				
**Marital status**						
Single	87 (32.3)	236 (27.8)	3.2	**Ref**	-	0.07
Divorced	30 (11.2)	62 (7.3)		**1.3**		
Married	121 (45.0)	456 (53.6)		**0.7**		
Widowed	31 (11.5)	96 (11.3)		**0.9**		
**ART regimen** **						
2NRTIs + 1NNRTI	245 (84.5)	785 (84.8)	0.7	-	-	0.69
2NRTIs + 1PI	33 (11.4)	94 (10.1)				
3NRTIs	12 (4.1)	47 (5.1)				
**Type of clinic**						
Religious	19 (6.6)	104 (11.2)	18.9	-	-	0.0001
Public	186 (64.1)	461 (49.8)				
NGO***	85 (29.3)	361 (39.0)				

* PLWHA: People Living With HIV/AIDS

** NRTI: Nucleoside Reverse Transcriptase Inhibitors; NNRTI: Non-Nucleoside Reverse Transcriptase Inhibitors; PI: Protease Inhibitors

*** NGO: Non Governmental Organization

During multivariate analysis, we included variables significant at p < 0.05 on bivariate analysis. Of the 1216 patients, 1119 (93%) had data for all relevant variables and were included in the model. In the model, hemoglobinemia was removed (p = 0.3). The following factors remained significantly associated with early lost to follow up of PLWHA on ART: age below 35 years (OR = 1.6; 95% CI: 1.2-2.2), female sex (OR = 1.8; 95% CI: 1.3-2.5), WHO stage III or IV (OR = 1.7; 95% CI: 1.3-2.2), existence of an opportunistic infection (OR = 2.3; 95% CI: 1.5-3.1), and follow-up in a public structure (OR 1.9; 95% CI: 1.2-3.3) (Table 3).

**Table 3:** Multivariate analysis of factors associated with lost to follow-up at ≤6 months compared to greater than 6 months among patients with human immunodeficiency virus (HIV) infection on antiretroviral therapy; Togo, 2008-2011

Variable	Odds ratio	95% CI	p-Value
Age < 35 years	1.6	1.2 - 2.2	**0.0008**
Female	1.8	1.3 - 2.5	**0.0002**
WHO stage 3 or 4	1.7	1.3 - 2.2	**0.0004**
Opportunistic infection	2.3	1.5 - 3.1	**<0.001**
**Type of HIV care clinic**			
Religious	Ref	**1**	-
Public	1.9	1.2 - 3.3	**0.02**
NGO*	1.1	0.6 - 1.9	**0.8**

* NGO: Non Governmental Organization

Among the 1216 patients, no telephone number was mentioned in the medical records of 212 patients and for 802 patients, the listed telephone number did not work. Of the remaining 202 patients, 114 patients (56%) were alive and 88 (44%) had died.

## Discussion

This study shows that the factors in Togo associated with early loss to follow of PLWHA on ART are age, gender, WHO stage of disease, existence of an opportunistic infection and type of clinic delivering HIV/AIDS care. Among the subset of patients for whom this information was available, we have demonstrated that a major reason for lost to follow-up is death; it is possible that a major reason we could not contact many people by telephone is because of death, and if this occurred, our mortality estimates will be conservative. In summary, lost to follow-up frequently follows severe disease, including death, and particularly among women and younger adults.

The proportion of patients lost to follow reported in Togo was significantly lower than the 13-40% reported by other studies in sub-Saharan Africa [4, 7]. This may have resulted from different time intervals used to define loss to follow: in our study this was 4 months, while other studies used values of 60 days [8], 6 months [2, 9] or 14 months [7]. A multicenter study in Africa, Asia, and Latin America recently recommended using 6 months as the standard definition to improve comparability [10].

The severity of disease also likely played a role in determining our results. The CD4 count in our study was less than 200 / mm3 for 76% of patients at the beginning of ART with a median count of 119/mm3. Thus, in Togo most patients begin ART once they meet the clinical criteria for AIDS. While this is consistent with other studies from sub-Saharan Africa [2, 11] it will not be true in other more affluent areas or possibly in Africa over time.

In multivariate analysis, five factors (age, sex, WHO stage III or IV, presence of opportunistic infections and type of HIV care center) were associated with early loss to follow up of PLWHA on ART. Our study could not determine further why these factors contributed to loss to follow-up. Three of the factors — WHO stage [12, 13], opportunistic infections [11, 14, 15], and among a subset death — are all consistent and illustrate that in our setting the most ill patients become lost to the medical care system. The other factors require more investigation to explain. Unlike our study, Ekouevi et al. [2] reported that in West Africa males were more likely than females to have low retention on ART. Early lost to follow up in young patients may be related to psychological denial of HIV infection or by the use of other types of treatment [16]. Use of a public clinic may reflect socioeconomic status with poorer patients more likely to be lost to follow-up.

In our study, 80% of patients whose phone numbers existed in their medical record could not be reached on these numbers. Previous studies have reported values of 35% [17] and 27% [18]. The explanation for this discrepancy, but may relate to socio-cultural issues, such as patients providing incorrect telephone numbers because of fear of stigmatization, denial of infection or a preference for traditional treatment. Alternatively, it could be that most patients whom we could not track had died, since this outcome had occurred for almost half of the patients whom we could track. Other studies support this theory: Yu et al [18] reported that 73% of all patients could be tracked, 50% had died and 23% were alive while a meta-analysis from sub-Saharan Africa reported that 40% of patients lost to follow-up had died [19].

The national STI/HIV/AIDS control program should take these results into account to strengthen the medical support of PLWHA in Togo, targeting young patients, those seeking care in public centers, and those with the most severe disease. For example, community health workers could actively track persons with HIV. Less expensively, health care providers could send SMS text messaging reminders, targeting high risk groups, a method that has worked in other settings [20, 21]. Most generally, to the extent that severe disease and mortality are key factors in loss to follow-up, improvements in clinical care — such as better laboratory monitoring, sustained access to ART, and increased patient education on the utility of modern medical interventions — may provide the best long-term solution.

### Limitations

As with many retrospective studies, data were missing from many patients, particularly for information collected during the phone follow-up component. Consequently, we likely did not fully evaluate some important risk factors. Additionally, data may have been incompletely recorded in medical records. This could have affected risk factor data (e.g., incorrect or missing CD4 counts) or outcome data (e.g., lack of accurate recording of death or change of residence for patients on ART). This is a necessary limitation for our study, where we aimed to determine risk factors for lost to follow-up in actual practice. While we could have performed a prospective study with periodic patient tracking (e.g., weekly) this design would have altered usual care seeking behaviors.

## Conclusion

Factors associated with the early loss to follow-up of PLWHA on ART in Togo were age, sex, clinical WHO stage III or IV, presence of opportunistic infection, and type of HIV care center; moreover, many patients lost to follow-up died. These results suggest specific groups that could be targeted with active case follow-up or SMS messaging.
